# High prevalence of persistent pain 6 months after arthroscopic subacromial decompression and/or acromioclavicular joint resection

**DOI:** 10.1051/sicotj/2019021

**Published:** 2019-06-18

**Authors:** Lone Dragnes Brix, Theis Muncholm Thillemann, Karen Toftdahl Bjørnholdt, Lone Nikolajsen

**Affiliations:** 1 Department of Anaesthesiology, Horsens Regional Hospital Sundvej 30 8700 Horsens Denmark; 2 Department of Orthopaedic Surgery, Aarhus University Hospital Palle Juul-Jensens Blvd. 99 8200 Aarhus N Denmark; 3 Department of Orthopaedic Surgery, Horsens Regional Hospital Sundvej 30 8700 Horsens Denmark; 4 Department of Anaesthesiology and Intensive Care, Aarhus University Hospital Palle Juul-Jensens Blvd. 99 8200 Aarhus N Denmark

**Keywords:** Shoulder arthroscopy, Persistent pain, Conditioned pain modulation, Postoperative pain

## Abstract

*Purpose*: The aims of this prospective study were to determine the prevalence of pain 6 months after arthroscopic subacromial decompression (ASD) and/or acromioclavicular joint resection (AC resection), to reveal causes of the pain, and to identify risk factors for persistent pain.

*Methods*: Preoperatively, patients were tested for their endogenous capacity to modulate pain and completed questionnaires concerning psychological vulnerability. Patients with pain 6 months after surgery were examined by an experienced orthopaedic surgeon to reveal any shoulder pathology responsible for the pain.

*Results*: Data from 101 patients were available for analysis 6 months after surgery. Thirty-six patients had persistent pain: 32 underwent examination by the surgeon who identified shoulder pathology in ten patients, but not in the remaining 22 in whom ongoing insurance case, unemployment, and a general tendency to worry were risk factors for persistent pain.

*Conclusion*: The prevalence of persistent pain 6 months after ASD and/or AC resection was 35.6% (95% CI 26.1–45.8%) and the proportion of patients with shoulder pathology was 9.9%. An association between ongoing insurance case, unemployment, general tendency to worry (t-STAI), and unexplained persistent pain 6 months after surgery was found.

## Introduction

Shoulder pain is one of the most common musculoskeletal complaints and leads to an increasing rate of outpatient arthroscopic shoulder surgery such as arthroscopic subacromial decompression (ASD) and acromioclavicular joint resection (AC resection) [1]. High success rates have been reported in the literature; however, the effectiveness of ASD and/or AC resection has been questioned as a fair amount of patients experience some degree of persistent symptoms e.g., persistent pain [2–6]. Only few studies have defined “failure of surgery” and they have reported failure rates of 16–25%, but these studies lack clinical follow-up examination in order to identify any shoulder pathology responsible for the pain [4, 5, 7–12].

Therefore, these high failure rates stress the importance of identifying preoperative risk factors for a poor outcome and persistent pain. Several preoperative risk factors for persistent pain have previously been identified in other types of surgery, including a decreased capacity of endogenous pain modulation [13] and psychological vulnerability [14–16]. However, to the best of our knowledge research to date has not yet investigated the potential association between the surgical procedures ASD and/or AC resection and persistent pain. Therefore, the aims of the present prospective study were (1) to determine the prevalence of persistent pain after ASD and/or AC resection, (2) to reveal shoulder pathology responsible for the pain by thorough follow-up examination of the patients, and (3) to investigate whether a preoperative decreased capacity of endogenous pain modulation and psychological vulnerability are risk factors for unexplained persistent pain. We hypothesized that at least 10% of patients would experience pain without shoulder pathology 6 months after surgery and that risk factors include preoperative decreased capacity of endogenous pain modulation and psychological vulnerability.

## Materials and methods

A prospective observational study with a 6-month follow-up was conducted. Based on the preoperative diagnosis and after obtaining signed informed consent, 150 patients scheduled for outpatient arthroscopic shoulder surgery (ASD and/or AC resection) were enrolled at the Day Surgery Unit at Horsens Regional Hospital. Primary exclusion criteria included age <18 years of age, shoulder surgery within the last year, Raynaud’s phenomenon, psychiatric illness, or inability to communicate in our language. Secondary exclusion criteria included more extensive surgery than planned, e.g., intraoperative rotator cuff repair, biceps tenodesis or labral repair, since a longer recovery period would be expected for these patients. The study was approved by the Data Protection Agency (1-16-02-213-14), the Committees in Health Research Ethics (1-10-72-103-14), and registered at ClinicalTrials.gov (NCT02924519).

### Postoperative care and treatment

Oral postoperative analgesic treatment was started before discharge from the post-anaesthesia care unit at the Day Surgery Unit and consisted of acetaminophen 1 g orally every 6 h up to 4 g daily, ibuprofen 400 mg orally three times daily. If ibuprofen was contraindicated, it was replaced by tramadol 50 mg orally four times daily. As rescue medication, patients were instructed in taking morphine 10 mg orally with a maximum of 60 mg per day. If morphine was contraindicated, it was replaced by 5 mg oxynorm with a maximum of 30 mg per day.

Postoperatively, patients were provided with written instructions about postoperative exercises including passive exercises and active exercises for the rotator cuff. Patients were told to follow the exercise programme to the best of their ability: use the arm freely and perform the exercises until pain threshold. The postoperative rehabilitation exercises in the first 3 months were self-monitored and patients were offered an examination with the physiotherapist 3 months after surgery.

### Persistent pain

We chose to define pain as being persistent postoperative pain if the following criteria were met: (1) pain 6 months after surgery, (2) average pain intensity within the last 2 weeks ≥ 3 on a numerical rating scale (NRS, from 0 = no pain to 10 = worst imaginable pain), and (3) confirmed impact of pain on the patient’s daily living (no/yes).

Patients, who fulfilled the criteria for persistent pain, were offered a thorough clinical examination by an experienced shoulder surgeon supplemented by diagnostic imaging, if relevant. The pain was classified as “unexplained persistent pain” if no shoulder pathology was responsible for the pain.

### Endogenous pain modulation

Preoperatively, on the day of surgery, a conditioned pain modulation test was performed to examine the endogenous pain modulation capacity of the patient. An efficient endogenous pain modulation capacity is important in order to cope with e.g., postoperative pain. The test is known as a “pain inhibits pain” paradigm and is typically assessed by recordings of pain elicited by a painful (test) stimulus before and during the application of another (conditioning) painful stimulus (e.g., cold water immersion) [17]. Thus, a patient was considered to have an efficient endogenous pain modulation capacity if the test pain was reduced during cold water immersion [17, 18].

In this study, pressure pain was used as test stimulus (obtained by using a handheld pressure algometer, Somedic Hörby, Sweden) and cold water immersion as conditioning stimulus (using a box containing ice water at 2 °C). In brief, pain elicited by 1.5 × pressure pain threshold was assessed before and during submersion of the contralateral hand into cold water. The pressure algometer was applied at two sites; one on the central part of supraspinatus muscle of the affected shoulder and one 10 cm proximal to patella on the central part of quadriceps muscle on the contralateral side. The test was performed by one of the authors, or in a few cases by a trained project nurse. The duration of the test was approximately 15 min with a maximum of 2 min in the cold water.

### Questionnaires

All patients completed six paper questionnaires before surgery. One questionnaire was developed by the authors for the purpose of this study and contained questions about shoulder pain [intensity was recorded on a Numeric Rating Scale (NRS, 0–10)], pain elsewhere (no/yes), and baseline characteristics (age, gender, BMI, level of education, marital status, ongoing insurance case, and employment).

The remaining five questionnaires were all validated in Danish and included two patient-reported outcome measures, i.e., the Western Ontario Rotator Cuff Index (WORC) [19] and the Single Assessment Numeric Evaluation (SANE) [20] as well as three psychological questionnaires: the State-Trait Anxiety FORM Y (STAI), Hospital Anxiety and Depression Scale (HADS), and Pain Catastrophizing Scale (PCS). The WORC index measures shoulder-specific quality of life and consists of 21 visual analogue scale items organized in five subscales: physical symptoms, sports/recreation, work, lifestyle, and emotions. The maximum score is 2100, with a higher score representing lower quality of life [19]. SANE is determined by the patient’s written response to the following question, “How would you rate your shoulder today as a percentage of normal on a 0% to 100% scale with 100% being normal?” [20]. In the present study, the 75% quartile was used for cut-off in the WORC score and the 25% quartile for cut-off in the SANE score. STAI is a 40-question multiple choice questionnaire; 20 questions address the general tendency to worry (t-STAI) and 20 questions address the temporary state (s-STAI) with scores from 1 to 4, with a maximum of 80. A cut point of 39–40 has been suggested to detect clinically significant symptoms of anxiety [21]. HADS consists of 14 questions regarding symptoms of anxiety and depression scored on a scale from 0 to 3, maximum 21, where scores in the range 8–10 show possible anxiety/depression and scores greater than 10 indicate severe anxiety/depression [22]. The PCS is a 13-question multiple choice questionnaire measuring catastrophizing on a scale from 0 to 4, with a maximum of 52. A PCS score between 20 and 30 indicates moderate risk for the development of chronicity, and a score above 30 implies high risk for developing chronicity [23].

### 6-month follow-up

Six months after surgery, patients received a postal questionnaire regarding shoulder pain. Patients with persistent pain 6 months after surgery were offered a thorough musculoskeletal examination. Prior to the examination, patients underwent anterior-posterior, outlet view, and AC joint X-ray examination of the affected shoulder. This was done to exclude major pathology such as insufficient bone resection of the acromial spur or AC joint, glenohumeral osteoarthritis, calcifications or other degenerative changes. Next, the affected shoulder was examined to reveal potential sources of pain (e.g., rotator cuff pathology, frozen shoulder, biceps pathology, neuritis, osteoarthritis, instability, and scapular dyskinesia), and the elbow and neck were examined with focus on potential sources of referred pain. Patients were referred to diagnostic imaging including MRI, CT and local block test if considered relevant by the surgeon: intraarticular block test (if a frozen shoulder was suspected), and subacromial block test (if subacromial pathology was suspected).

### Statistical analysis

Data were entered into Excel and exported to Stata software version 15.0 (StataCorp, TX, USA), in which statistical analyses were performed.

A sample size of 140 patients was required to detect an expected prevalence of unexplained persistent pain of 10% (desired precision of 5% and a confidence interval of 95%). We chose to enrol 150 patients to allow for drop-outs. Results were presented as either mean ± standard deviation (parametric data) or as frequencies or medians with interquartile range (IQR) (non-parametric) as appropriate. All *p*-values were two-tailed and those below 0.05 were considered significant.

Risk factors for unexplained pain were identified using multivariable logistic regression models. All independent risk factors were assessed in univariable logistic models to estimate odds ratio (OR) with a significance level <5% including the following variables: age, gender, BMI, level of education, ongoing insurance case, employment, pain elsewhere, type of surgery, pain intensity in the shoulder, the conditioned pain modulation test response, WORC score, SANE score, the PCS, HADS and STAI.

## Results

**Figure 1:**
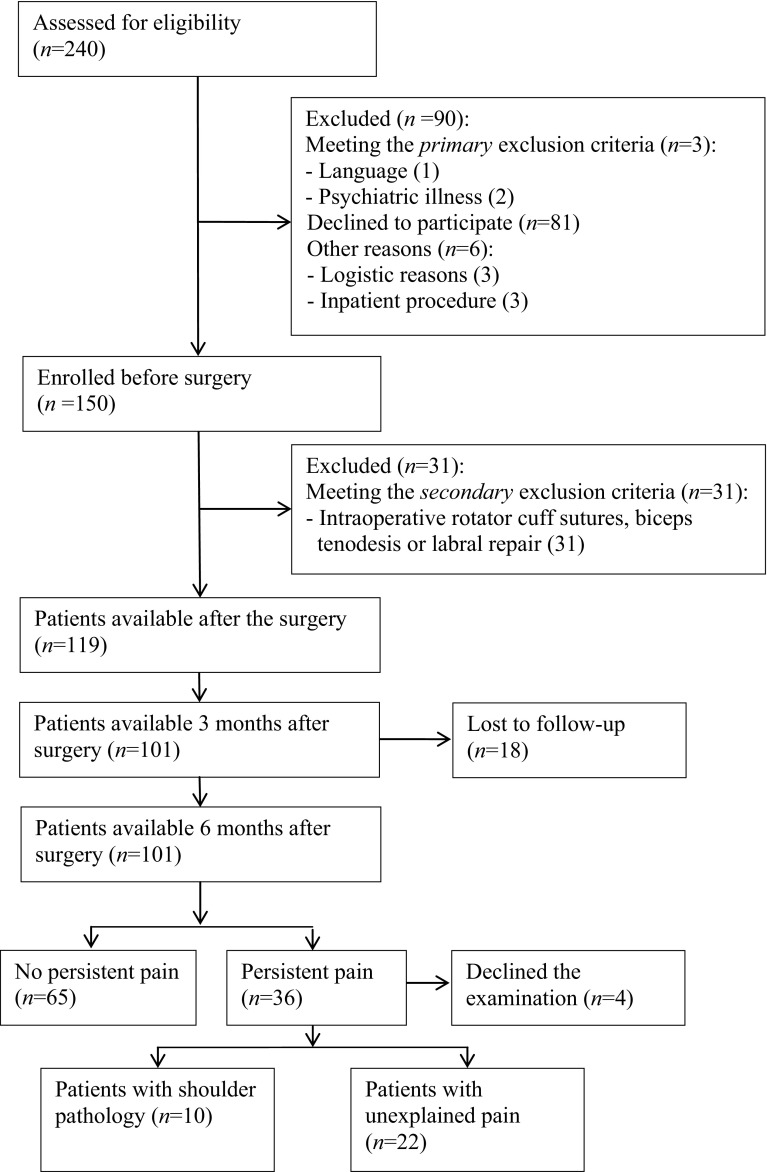
Flow chart.

A total of 240 patients were assessed for eligibility from October 2014 to June 2016. One-hundred-and-fifty patients agreed to participate and were enrolled in the study, 31 patients had to be excluded after surgery due to more extensive surgery than planned. In all, 119 participants were eligible and consenting. Forty-three patients underwent ASD, 20 patients underwent AC resection, and 56 patients underwent ASD with AC resection. All surgical procedures were performed by the same four experienced surgeons with specialty training in shoulder surgery. Eighteen patients did not respond to the 6-month questionnaire, leaving data from 101 patients available for analysis (Figure 1). Baseline characteristics are shown in Table 1.

**Table 1:** Patient characteristics.

Baseline characteristics	The total cohort	No persistent pain	Persistent pain	*p*-value
	*n* = 119	*n* = 65	*n* = 22	
Age in years, mean (*SD*)	56 (10.4)	59 (10.2)	54 (8.8)	0.04
Gender, *n* (% of total)				
Female	61 (51.3)	33 (50.8)	14 (63.6)	0.30
BMI (kg/m^2^), mean (*SD*)	26.8 (4.3)	27 (4.1)	28 (5.9)	0.56
Missing	5	0	0	
Level of education, *n* (% of total)				
Low level of education	91 (76.5)	38 (58.5)	17 (77.3)	0.29
High level of education	27 (22.7)	27 (41.5)	5 (22.7)	
Missing	1 (0.8)	0	0	
Marital status, *n* (% of total)				
Single/widowed	95 (79.8)	10 (15.4)	4 (18.2)	0.76
Cohabitants/married	23 (19.3)	55 (84.6)	18 (81.8)	
Missing	1 (0.8)	0	0	
Employment, *n* (% of total)				
No	23 (19.3)	7 (10.8)	10 (45.5)	<0.01
Yes	66 (55.5)	36 (55.4)	9 (40.9)	
Retired	25 (21.0)	20 (30.8)	3 (13.6)	
Missing	5 (4.2)	2 (3.1)	0	
Insurance case, *n* (% of total)				
Missing	14 (11.8)	2 (3.1)	7 (31.8)	<0.01
Pain elsewhere	1 (0.8)	0	0	
No	33 (27.7)	17 (26.2)	2 (9.1)	0.09
Yes	85 (71.2)	48 (73.8)	20 (90.1)	
Missing	1 (0.8)	0	0	
Shoulder-specific parameters				
WORC, mean (*SD*)	1284 (402)	1227 (392)	1455 (285)	0.01
SANE, mean (*SD*)	51 (18.4)	51 (19.1)	48.5 (13.9)	0.53
Worst pain, median [range]	7 [0–10]	7 [0–10]	7 [3–10]	0.29
24 h average pain, median [range]	5 [0–10]	5 [0–10]	5 [2–8]	0.67
Type of surgery, *n* (% of total)				
ASD	43 (36.1)	25 (38.5)	8 (36.4)	0.03
AC resection	20 (16.8)	6 (9.2)	7 (31.8)	
ASD and AC resection	56 (47.1)	34 (52.3)	7 (31.8)	
Expected outcome after surgery				
Average pain median [range]	7 [0–10]	7 [1–10]	7.5 [3–10]	0.26
Return to work/daily living*, n* (% of total)				
<4 weeks	41 (34.5)	23 (35.4)	9 (40.9)	0.68
1–2 months	40 (33.6)	28 (43.1)	4 (18.2)	
3–4 months	29 (24.4)	10 (15.4)	8 (36.4)	
5–6 months	3 (2.5)	1 (1.5)	0	
>6 months	2 (1.7)	1 (1.5)	0	
Missing	4 (3.4)	2 (3.1)	1 (4.5)	
Conditioned pain modulation				
PPT shoulder, mean (*SD*)	272 (140)	273 (140)	290 (110)	0.65
PPT thigh, mean (*SD*)	477 (226)	455 (217)	530 (207)	0.18
CPM shoulder, *n* (%)				
Inefficient	13 (10.9)	7 (10.8)	1 (4.5)	0.33
Efficient	79 (66.4)	45 (69.2)	18 (81.8)	
Missing	27 (22.7)	13 (20.0)	3 (13.6)	
CPM thigh, *n* (%)				
Inefficient	9 (7.6)	6 (9.2)	0	0.33
Efficient	80 (67.2)	45 (69.2)	17 (77.3)	
Missing	30 (25.2)	14 (21.5)	5 (22.7)	
Psychological parameters				
PCS total, *n* (%)				
Low risk	67 (56.3)	37 (56.9)	11 (50)	0.69
Moderate risk	31 (26.1)	18 (27.7)	8 (26.4)	
High risk	21 (17.6)	10 (15.4)	3 (13.6)	
HADS-a, *n* (%)				
Low risk	97 (81.5)	54 (83.1)	17 (77.3)	0.06
Moderate risk	13 (10.9)	6 (9.2)	3 (13.6)	
High risk	9 (7.6)	5 (7.7)	2 (9.1)	
HADS-d, *n* (%)				
Low risk	105 (88.2)	57 (87.7)	19 (86.4)	0.13
Moderate risk	8 (6.7)	5 (7.7)	1 (4.5)	
High risk	6 (5.0)	3 (4.6)	2 (9.1)	
s-STAI, *n* (%)				
Low risk	83 (69.7)	47 (72.3)	12 (54.5)	0.12
High risk	36 (30.3)	18 (27.7)	10 (45.5)	
t-STAI, *n* (%)				
Low risk	89 (74.8)	53 (81.5)	11 (50.0)	<0.01
High risk	30 (25.2)	12 (18.5)	11 (50.0)	

BMI, body mass index; WORC, Western Ontario Rotator Cuff Index; SANE, Single Assessment Numeric Evaluation; ASD, arthroscopic subacromial decompression; AC resection, acromioclavicular joint resection; CPM, conditioned pain modulation STAI, State-Trait Anxiety FORM Y; HADS, Hospital Anxiety and Depression Scale; PCS, Pain Catastrophizing Scale.

Separate characteristics for patients with shoulder pathology (*n* = 10) and the four patients who declined to have a follow-up examination 6 months after surgery are not shown. The 18 non-responders had undergone ASD (*n* = 4), AC resection (*n* = 4) and ASD with AC resection (*n* = 10) and were significantly younger (*p* = 0.03), but did not differ in regard to gender, psychological characteristics, or conditioned pain modulation response. A patient was considered to have an efficient endogenous pain modulation capacity if the test pain was reduced during cold water immersion.

Three months after surgery 73 out of 101 patients (72.3%) accepted the examination with the physiotherapist and 6 months after surgery 36 out of 101 patients (35.6%, 95% CI: 26.4–45.8%) had persistent pain; 65 patients (64.4%) had no pain (Figure 1). The 36 patients with pain were offered an examination by the orthopaedic surgeon; four patients declined of whom two had undergone ASD and two ASD with AC resection. Shoulder pathology was found in 10 out of the 32 patients with pain (9.9%), including insufficient bone resection of the acromion or AC joint (*n* = 4), frozen shoulder (*n* = 1), calcifying tendinitis (*n* = 1), cervical intervertebral disc protrusion (*n* = 1), AC joint arthritis (*n* = 1), os acromiale (*n* = 1), and multidirectional shoulder instability (*n* = 1). The distribution of the ten patients with an explained reason for the pain was as follows: ASD (*n* = 4), AC resection (*n* = 3), and ASD with AC resection (*n* = 3). The ten patients with shoulder pathology and the four patients who declined having an examination were excluded from further analysis, leaving 22 patients (21.8%, 95% CI 14.2–31.1%) with unexplained persistent pain. The 22 patients with unexplained persistent pain had undergone ASD (*n* = 8), AC resection (*n* = 7), and ASD with AC resection (*n* = 7).

The conditioned pain modulation was tested in 95 out of 101 patients. The number of patients with an inefficient endogenous pain inhibition was similar in patients with no persistent pain and unexplained persistent pain; *n* = 19; 29.2% vs. *n* = 4; 18.2%; *p* = 0.26 (shoulder) and *n* = 4; 6.2% vs. *n* = 3; 13.6%; *p* = 0.36 (thigh), respectively.

**Figure 2:**
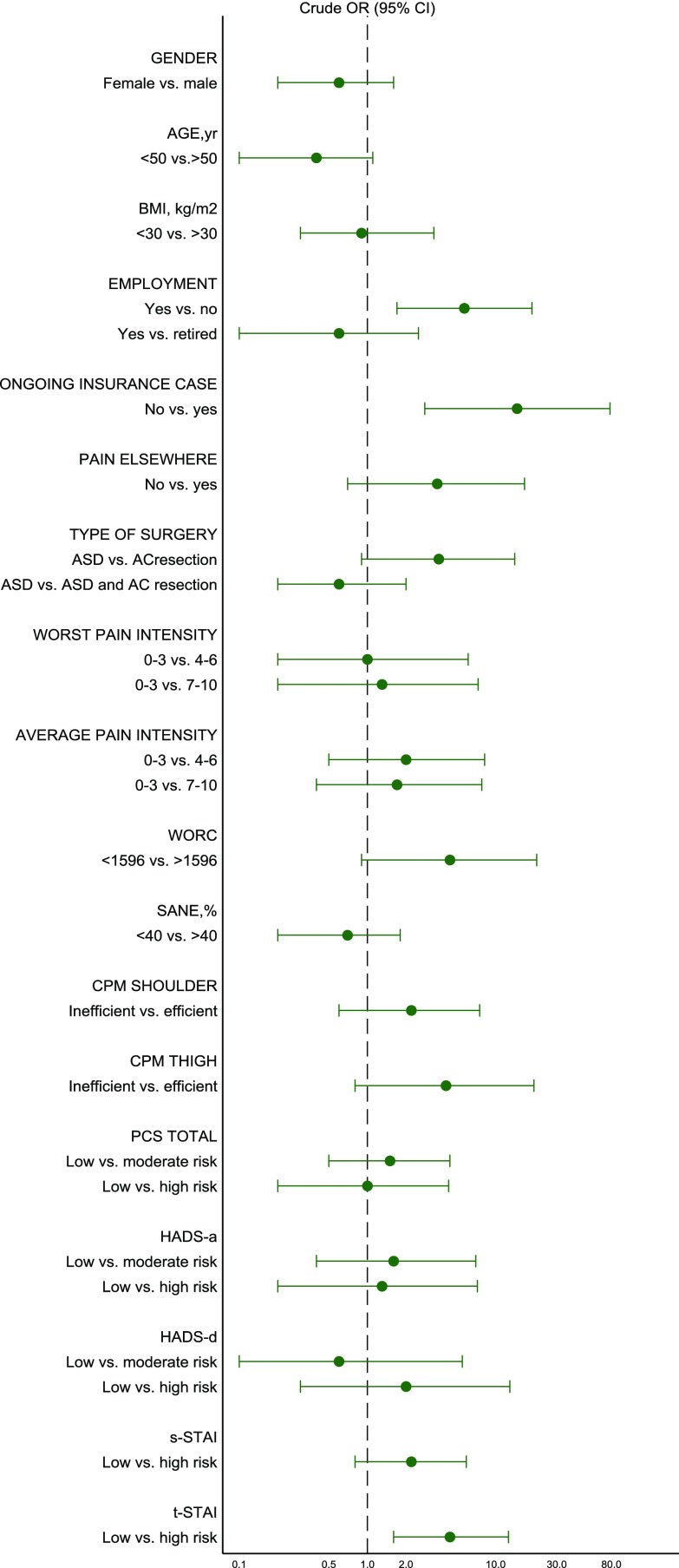
Preoperative risk factors for unexplained persistent pain (univariable).

Out of several potential preoperative risk factors including age, gender, BMI, level of education, ongoing insurance case, employment, pain elsewhere, pain intensity in the shoulder, WORC score, SANE score, the PCS, HADS, STAI, and the conditioned pain modulation test response, the univariable analysis (Figure 2) showed an association between unexplained persistent pain and the following independent risk factors: Ongoing insurance case OR 14.7 (95% CI 2.8–78.0), unemployment OR 5.5 (95% CI 1.7–19.2), and tendency to worry (t-STAI score > 40) OR 4.4 (95% CI 1.6–12.6). These variables were included as covariates in the multivariable logistic regression model (Table 2).

**Table 2:** Preoperative risk factors for unexplained persistent pain (multivariable).

Preoperative variables	Multivariable	
	OR	95% CI
Employment		
Yes		
No	7.4	1.9–29.3
Retired	0.6	0.1–3.3
Ongoing insurance case		
No		
Yes	17.7	2.8–110.1
t-STAI		
Low risk		
High risk	3.4	1.0–11.9

OR, odds ratio; CI, confidence interval; STAI, State-Trait Anxiety FORM Y.

## Discussion

In this study, we found that 35.6% (95% CI 26.4–45.8%) of patients still report pain 6 months after surgery. Thus, our findings are in accordance with the results from a retrospective study including 108 patients in which as many as 31 patients (29%) had >3 on a visual analogue scale score 6 months after ASD surgery [24]. Other studies have reported failure rates of 16–25% [4, 5, 7–12]. However, these studies either lack a clear definition of persistent pain or did not report intensity of pain using e.g., NRS or VAS. Instead they have mainly used shoulder-specific questionnaires (DASH, OSS and WORC) as outcome measures for improvement of the shoulder after ASD and/or AC resection.

At follow-up examination, we identified shoulder pathology responsible for the pain in ten patients (9.9%). The examination revealed many causes of persistent pain, which remind us of more careful preoperative diagnostics and the necessity of follow-up after surgery, targeted those with persistent pain, to uncover remaining pathology. The clinical examination is a strength of our study compared to the studies relying on follow-up by patient reports alone. To optimize the use of healthcare resources this could be done by screening patients using a questionnaire survey 6 months after surgery.

Only a few other studies have examined patients after ASD and/or AC resection. In an ASD study including 114 patients, 25% reported that the outcome was unsatisfactory 19 months (12–40 months) after surgery and they were offered a follow-up examination in which the authors were able to identify a medical reason for the failure in 19 patients (16.7%) [10]. However, due to the long duration between surgery and follow-up examination, the patients may have acquired a new shoulder disorder.

We evaluated several potential independent risk factors of unexplained persistent postoperative pain including a preoperative decreased capacity of endogenous pain modulation and psychological vulnerability. We were unable to demonstrate that patients with unexplained persistent pain had a less inefficient endogenous pain modulation capacity than patients without persistent pain [13, 25]. This result is in line with results from another prospective study where 73 patients scheduled for arthroscopic shoulder surgery underwent a conditioned pain modulation test using a suprathreshold heat pain response as test stimulus opposite to our pressure pain [26]. The negative findings in both studies are likely to be related to the high preoperative pain intensity which may have altered the sensibility of the central nervous system (endogenous pain modulation capacity). Other types of psychophysical tests may therefore be of interest in future studies in relation to prediction of persistent postoperative pain in patients undergoing orthopaedic surgery.

We found an association between ongoing insurance case, unemployment, and general tendency to worry (t-STAI) with unexplained persistent pain 6 months after surgery. Previous studies have shown that patients with workers’ compensation have lower shoulder-specific measures (e.g., DASH) and general health measures before and after surgery and have worse outcomes [24, 27, 28]. Even though the underlying factors for the association between a poor outcome and an ongoing insurance case is not fully understood, it is important to be especially aware of patients with ongoing insurance cases.

We expected to find an association between psychological vulnerability parameters (HADS, PCS and STAI) and unexplained persistent pain, however, except for the association between unexplained persistent pain and high t-STAI score, present results found no associations between unexplained persistent pain and psychological vulnerability parameters. These findings are in contrast with a retrospective ASD study where an association between persistent pain and depression was found [8]. A prospective study with recordings of patients’ vulnerability both before and after surgery is needed.

The study has a few shortcomings. Firstly, the sample size was not reached after 6 months; this reduced power in the regression, but the estimate of prevalence still had a reasonable confidence interval. Secondly, a longer follow-up period would have provided additional information, since studies have suggested that patients undergoing orthopaedic surgery, in particular shoulder surgery, have longer convalescence compared to patients undergoing e.g., abdominal and gynaecological surgical procedures [29]. However, 6 months is beyond the normal healing period for ASD and AC resection. Thirdly, it cannot be ruled out that the lack of supervision of a skilled physiotherapist during the postoperative rehabilitation exercises may contribute to the prevalence of unexplained persistent pain six, and this must be taken into account in future studies. Fourthly, it would have been of interest to assess analgesic consumption before and after surgery. Finally, we cannot rule out residual confounding of risk factors.

## Conclusions

In conclusion, the prevalence of persistent pain 6 months after ASD and/or AC resection was 35.6% (95% CI 26.1–45.8%) and the proportion of patients with shoulder pathology was 9.9%.

Furthermore, an association between ongoing insurance case, unemployment, general tendency to worry (t-STAI), and unexplained persistent pain 6 months after surgery was found. However, the present study was unable to demonstrate that patients with unexplained persistent pain had a more inefficient endogenous pain modulation capacity than patients without persistent pain.
